# Comparative analysis of small molecule and growth factor-derived human induced pluripotent stem cell-derived hepatocyte-like cells

**DOI:** 10.3389/fcell.2025.1594340

**Published:** 2025-06-26

**Authors:** Faizal Z. Asumda, Shadia Alzoubi, Kiyasha Padarath, Kimya Jones, Ravindra Kolhe, Ashis Kumar Mondal, Ahmet Alptekin, Wenbo Zhi, Tae Jin Lee, Robert C. Huebert, Nathan P. Staff, Lewis R. Roberts, Lindsey A. Kirkeby

**Affiliations:** ^1^ Medical College of Georgia-Augusta University Department of Pediatrics, Augusta, GA, United States; ^2^ Medical College of Georgia-Augusta University Department of Pathology, Augusta, GA, United States; ^3^ Medical College of Georgia-Augusta University Vascular Biology Center, Augusta, GA, United States; ^4^ Medical College of Georgia-Augusta University Center for Biotechnology and Genomics, Augusta, GA, United States; ^5^ Mayo Clinic Division of Gastroenterology and Hepatology, Rochester, MN, United States; ^6^ Mayo Clinic Department of Neurology Rochester, Rochester, MN, United States; ^7^ Mayo Clinic Center for Regenerative Biotherapeutics, Rochester, MN, United States

**Keywords:** hepatocytes, hepatocyte-like cells, induced pluripotent stem cells, growth factors, small molecules

## Abstract

The growth factor and small molecule protocol are the two primary approaches for generating human induced pluripotent stem cell-derived hepatocyte-like cells (iPSC-HLCs). We compared the efficacy of the growth factor and small molecule protocols across fifteen different human iPSC lines. Morphological assessment, relative quantification of gene expression, protein expression and proteomic studies were carried out. HLCs derived from the growth factor protocol displayed mature hepatocyte morphological features including a raised, polygonal shape with well-defined refractile borders, granular cytoplasm with lipid droplets and/or vacuoles with multiple spherical nuclei or a large centrally located nucleus; significantly elevated hepatocyte gene and protein expression including AFP, HNF4A, ALBUMIN, and proteomic and metabolic features that are more aligned with a mature phenotype. HLCs derived from the small molecule protocol showed a dedifferentiated, proliferative phenotype that is more akin to liver tumor-derived cell lines. These experimental results suggest that HLCs derived from growth factors are better suited for studies of metabolism, biotransformation, and viral infection.

## Introduction

Human induced pluripotent stem cell (iPSC)-derived hepatocyte-like cells (HLCs) present a unique opportunity for translational medicine (Corbett and Duncan, 2019; Tricot et al., 2022; Lu et al., 2015; Luo et al., 2023; Ardisasmita et al., 2022; Blackford et al., 2019). The two main protocols for producing induced pluripotent stem cell-derived hepatocyte-like cells (iPSC-HLCs) are the small molecule (SM) (Siller et al., 2015; Mathapati et al., 2016; Asumda et al., 2018; Gao et al., 2020; Du et al., 2018; Pan et al., 2022) and growth factor (GF)-based (Xia et al., 2017; Carpentier et al., 2016; Si-Tayeb et al., 2010) approaches. Generating HLCs with a level of hepatic differentiation and function that is synonymous with primary human hepatocytes (PHHs) is critical for regenerative and translational stem cell research. HLCs are an excellent patient and tissue specific disease model for many liver diseases including those with an underlying genetic etiology. Human iPSC derived HLCs are especially useful for metabolic and non-metabolic genetic disease modeling because pathogenic variants in the patient are preserved in both the fibroblast and reprogrammed iPSC lines. The resulting differentiated HLCs recapitulate the phenotype of the gene variant (Zhang et al., 2021; Gurevich et al., 2020; Duwaerts et al., 2021; Mukhopadhyay et al., 2024; Thiesler et al., 2016; Berger et al., 2016; Pournasr and Duncan, 2017). PHHs are the “gold standard” for *in vitro* modeling of the liver but cost, accessibility and variability between batches is a major drawback (Ardisasmita et al., 2022; Usynin and Panin, 2008; Aizarani et al., 2019). Immortalized hepatic tumor-derived cell lines are an alternative but these cell lines are fundamentally not expected to function in the same manner as a healthy human hepatocyte from a metabolic and physiologic standpoint. Immortalized hepatic tumor cell lines are expected to have metabolic changes that is designed to sustain their energy requirements, proliferative capacity and survival. They are more likely to have tumor related alterations in basic metabolic and physiologic pathways such as energy production and consumption (Glycolysis, Krebs cycle, lipid and amino acid metabolism) (Todisco et al., 2019; Ballester et al., 2019; Wiśniewski et al., 2016; Yang et al., 2024; Sajnani et al., 2017; Fekir et al., 2019; Zhang et al., 2019; Ciccarone et al., 2017). The current approach for the generation of HLCs from iPSCs mimics three key *in vivo* embryonic liver developmental stages: endoderm, hepatoblast and hepatocyte maturation (Luo et al., 2023; Ardisasmita et al., 2022; Blackford et al., 2019; Siller et al., 2015; Mathapati et al., 2016; Asumda et al., 2018; Gao et al., 2020; Du et al., 2018; Pan et al., 2022; Xia et al., 2017; Carpentier et al., 2016; Si-Tayeb et al., 2010; Zhang et al., 2021; Gurevich et al., 2020; Duwaerts et al., 2021; Mukhopadhyay et al., 2024). Both the SM and GF protocols have been shown to produce iPSC-HLCs with characteristics of PHHs. These include but are not limited to PHH gene and protein expression, enzyme activity of cytochromes P450 (CYPs), urea synthesis, glycogen storage, and ALBUMIN secretion (Luo et al., 2023; Ardisasmita et al., 2022; Blackford et al., 2019; Siller et al., 2015; Mathapati et al., 2016; Asumda et al., 2018; Gao et al., 2020; Du et al., 2018; Pan et al., 2022; Xia et al., 2017; Carpentier et al., 2016; Si-Tayeb et al., 2010; Zhang et al., 2021; Gurevich et al., 2020; Duwaerts et al., 2021; Mukhopadhyay et al., 2024). The limitation of iPSCs for *in vitro* modeling is the variation in line differentiation capacity and outcomes. For this reason, we are utilizing 15 different iPSC lines in these studies to ensure reproducibility of the two differentiation protocols across multiple lines. To help understand the underlying rewiring of iPSCs during differentiation to HLCs, we utilize proteomic analysis to help identify the key factors involved in iPSC response to stimuli.

In comparison to the growth factor protocol, the small molecules appear to be a cheaper and simpler logistical approach for producing HLCs, but an objective comparison demonstrates that a larger number of components are required in the SM protocol (Siller et al., 2015; Mathapati et al., 2016; Asumda et al., 2018). The simplified GF approach requires a single GF component (HGF) beyond the endoderm stage (Xia et al., 2017; Carpentier et al., 2016; Si-Tayeb et al., 2010). Our experience with both protocols has been that, while both produce HLCs with overlapping features of PHHs, there are striking differences in the phenotype of HLCs produced from each protocol. For this reason, an objective comparative analysis of the two protocols required to help direct the field. In this present study, we utilized a simple and straightforward GF protocol alongside the most widely published SM approach. We tested the hypothesis that HLCs derived from GFs are more physiologically and metabolically synonymous with healthy PHHs and are a better fit for modeling of healthy adult mature liver hepatocytes.

## Methods and materials

### Small molecules, chemicals, growth factors and antibodies

STEMdiff^TM^ Definitive Endoderm Kit (05,111), mTesR (85,850) and ReLeSR (100–0,483) were from Stem Cell Technologies (Vancouver, Canada). Hepatocyte Growth Factor (HGF) (294HGN100) was from R&D Systems, Minneapolis, MN. Dimethyl sulfoxide (DMSO) (D5879) was from Sigma Aldrich (St. Louis, MO). Dihexa (N-hexanoic-Tyr-Ile-(6) aminohex-anoic amide) (DC9760) was from DC Chemicals (Pudong District, Shanghai, China). Stemolecule CHIR99021 (04–0,004) was from Stemgent (Lexington, MA). Selective p160ROCK Inhibitor Y-27632 (1,254) was from Tocris (Minneapolis, MN). L-15 Medium (L1518), 2-Mercaptoethanol (M6250), Tryptose Phosphate Broth (T8159), Hydrocortisone-21- hemisuccinate (H4881), Sodium-L-Ascorbate (A4034), and Dexamethasone (D4902) were from Sigma (St. Louis, MO). RPMI/B27 medium (61,870,036), B27 supplement (17,504,044), GlutaMax supplement (35,050,061), Insulin- Transferrin-Selenium supplement (41,400,045), Knock Out DMEM (10,829,018), KnockOut Serum Replacement (10,828,028), Fetal Bovine Serum (A3840002), MEM Non-Essential Amino Acids Solution (11,140,050), Geltrex (A1413302), Dulbecco’s phosphate-buffered saline, calcium and magnesium free DPBS^−/−^(14,190,144), ProLong Gold Antifade Mountant with DAPI (P36935), Trypsin (90,057), Trizol Reagent (15,596,026) were from Thermo Fisher Scientific (Rockford, IL).

Human Serum ALBUMIN ELISA Kit (1,190) was from Alpha Diagnostic International (San Antonio, TX). Urea assay kit (ab83362) and Human alpha 1 Antitrypsin ELISA Kit (ab108799) were from Abcam (Cambridge, MA). Periodic Acid-Schiff (PAS) Kit (395B-1 KT) was from Sigma (St. Louis, MO). Antibodies against GAPDH (sc-47724), OCT4 (sc-5279), SOX2 (sc-365964), KLF4 (sc-166238), SOX17 (sc-130295), NANOG (sc-293121), SSEA-1 (sc-21702), TRA-160 (sc-21705), TRA-180 (sc-21706), FOXA2 (sc-271103), CXCR4 (sc-53534), HNF4A (sc-374229), A1AT (sc-166018), ALBUMIN (sc-271605) and AFP (sc-80464) were from Santa Cruz Biotechnology, Inc (Santa Cruz, CA). Secondary HRP-conjugated antibody (62–6,520), FITC-conjugated anti-rabbit (A21206), anti-mouse (A11059), TRITC-conjugated anti-rabbit (R37117) and anti-mouse (A11005) antibodies were from Thermo Fisher Scientific (Rockford, IL). iScript cDNA Synthesis Kit (1,708,891), iTAQ Universal SYBER Green Supermix (1,725,124) were from Bio-Rad (Hercules, CA). RIPA Buffer (AAJ63324EQE) was from Fisher Scientific (Hampton, NH). Pierce^TM^ BCA Protein Assay Kit (23,227) from Thermo Fisher Scientific (Rockford, IL). Commercial Primary human hepatocytes (5,200) were from ScienCell Research Laboratories (Carlsbad, CA). Liver tumor-derived cell lines (HUH7, HEP3B, HEPG2, SNU398) were a kind gift from Dr. Lewis Roberts at the Mayo Clinic (Rochester, MN).

#### Human pluripotent stem cell culture

A total of 15 healthy human iPSC lines were sourced from the Mayo Clinic Biotrust (Rochester, Minnesota) and utilized for these experiments. Approval was obtained from the Mayo Clinic Center for Regenerative Biotherapeutics Biotrust Oversight Group (IRB # 14–001464) and by the Medical College of Georgia-Augusta University Human Stem Cell Research Committee. Human iPSCs were adapted onto feeder-free conditions by plating on Geltrex (Thermo Fisher Scientific, Rockford, IL) coated plates, and fed daily with mTeSR medium (Stem Cell Technologies, Vancouver, Canada) at 37°C 5% CO_2_. The mTeSR medium was supplemented with 10 µM of p160ROCK inhibitor Y-27632 (Tocris, Minneapolis, MN) during the first 24 h after thawing. The cGMP, enzyme-free human pluripotent stem cell selection and passaging reagent ReLeSR (Stem Cell Technologies, Vancouver, Canada) was used for passaging. Cells were characterized as previously described (Asumda et al., 2018). Pluripotency was monitored by immunostaining for the pluripotency and iPSC markers OCT4, SOX2, NANOG, KLF4, Tra- 161, Tra- 181, SSEA4 and qRT-PCR for OCT4, SOX2, NANOG, c-Myc, KLF4 and REX1.

#### In vitro differentiation of iPSCs into hepatic cells

Human iPSCs were differentiated into HLCs using a growth factor (GF)-based and small molecule-based protocol. The growth factor protocol is adapted primarily from (Xia et al., 2017) with minor modifications in timing and concentrations. The small molecule protocol is adapted primarily from (Siller et al., 2015; Mathapati et al., 2016; Asumda et al., 2018; Gao et al., 2020; Du et al., 2018; Pan et al., 2022).

#### Definitive endoderm formation

For small molecule Definitive Endoderm (DE) differentiation, human iPSCs cultured in 6-well dishes to 60% confluence were washed sequentially with DPBS^−/−^ and DMEM/FI2 (Thermo Fisher Scientific). Human iPSCs were cultured in RPMI/B-27 (Thermo Fisher Scientific) with insulin and 6uM CHIR99021 (Stemgent) for 72 h at 37°C 5% CO2 with daily media changes. CHIR99021 was removed after 72 h and DE cells were cultured in RPMI/B27 + Insulin for 24 h (Siller et al., 2015; Mathapati et al., 2016; Asumda et al., 2018; Gao et al., 2020; Du et al., 2018; Pan et al., 2022).

For growth factor Definitive Endoderm (DE) differentiation, human iPSCs cultured in 6-well dishes to 60% confluence were washed sequentially with DPBS^−/−^ and DMEM/FI2 medium (Thermo Fisher Scientific). DE was induced using the STEMdiff^TM^ Definitive Endoderm Kit (Stem Cell Technologies). Briefly, cells are cultured with the STEMdiff^TM^ definitive endoderm basal medium with supplements A and B for 24h followed by STEMdiff^TM^ definitive endoderm basal medium with supplement B for 48h with daily medium change (Xia et al., 2017). The STEMdiff^TM^ DE kit is based primarily on human activin A and Wnt3a (Xia et al., 2017).

#### Hepatoblast specification

For small molecule hepatoblast specification, DE cells were washed with DPBS^−/−^ and knockout DMEM (Thermo Fischer Scientific) and sequentially cultured with hepatoblast specification medium without passaging. Small molecule hepatoblast specification medium contained Knockout DMEM (Thermo Fisher Scientific), 1% DMSO (Sigma), 5 mM GlutaMAX (Thermor Fisher Scientific), 1% non-essential amino acids (Thermo Fisher Scientific) and 100 µM 2-mercaptoethanol (Sigma). DE cells were cultured in hepatoblast specification media at 37 °C, 5% CO_2_ for 6 days with media changes every 48 h (Siller et al., 2015; Mathapati et al., 2016; Asumda et al., 2018; Gao et al., 2020; Du et al., 2018; Pan et al., 2022).

For growth factor hepatoblast specification, DE cells were washed with DPBS^−/−^ and DMEM/F12. Without passaging, DE cells were sequentially cultured with hepatoblast specification medium (DMEM/F12, 10% KOSR, 1% Glutamine, 1% non-essential amino acids, 1% Penicillin/Streptomycin; Thermo Fischer) containing 100 ng/mL of hepatocyte growth factor (HGF, R&D Systems), 1% dimethyl sulfoxide (DMSO; Sigma Aldrich) for 6 days with fresh medium every 48 h (Xia et al., 2017).

### Hepatocyte-like cell maturation and maintenance

For small molecule HLC maturation, hepatoblasts were incubated in maturation medium containing L-15 Leibovitz medium (Thermo Fisher Scientific), 100 mM Dihexa (DC Chemicals), 3% Insulin-Transferrin-Selenium supplement (Thermo Fisher Scientific), 10% Fetal Bovine Serum (Thermo Fischer Scientific), 2 mM GlutaMAX (Thermo Fisher Scientific), 10% Tryptose Phosphate Broth (Thermo Fisher Scientific), 10 µM hydrocortisone-21-hemisuccinate (Sigma), 100 nM Dexamethasone (Sigma) and 50 μg/mL Sodium-L-Ascorbate (Sigma). Hepatoblasts were incubated in hepatic maturation media at 37 °C, 5% CO_2_ for 14 days with media changes every 48 h (Siller et al., 2015; Mathapati et al., 2016; Asumda et al., 2018; Gao et al., 2020; Du et al., 2018; Pan et al., 2022). All small molecules were resuspended in DMSO. Small molecule derived HLCs were maintained with medium consisting of the hepatocyte maturation medium devoid of DMSO and Dihexa.

For the growth factor HLC maturation, hepatoblasts were cultured for 14 days in hepatoblast specification medium supplemented with 10^–7^ M dexamethasone (Sigma Aldrich).

Growth factor-derived HLCs were maintained in medium consisting of William’s E Medium, 10% FBS, 1% GlutaMax, 1% NEAA, 1% Penicillin/Streptomycin (Thermo Fisher Scientific), 1% Insulin-Transferrin-Selenium supplement (Sigma), 10^–7^ M dexamethasone (Sigma Aldrich). Maintenance medium is devoid of DMSO and HGF (Xia et al., 2017).

#### Immunocytochemistry

Immunocytochemistry staining was completed as previously described (Asumda et al., 2018). Briefly, cells were grown on glass cover slips, washed with PBS, fixed with 2% paraformaldehyde for 10min at room temperature, quenched with 100 mmol/L glycine (pH 7) for 5 min, permeabilized with 0.1% Triton-X- 100 for 5min, blocked with 1% BSA in PBS for 20min at room temperature, and sequentially incubated with respective primary antibodies diluted in blocking solution (1% BSA in PBS) at 4 °C overnight and then with appropriate fluorophore labeled secondary antibodies diluted in blocking solution (1% BSA in PBS) for 1 h at 37 °C in the dark. Controls were incubated with secondary antibodies only. Coverslips were mounted onto glass microscope slides with Prolong Gold Antifade with DAPI (Thermo Fisher Scientific). Confocal images of immunostained cells were obtained using ×60 oil objective on a Zeiss 780 Inverted Confocal Microscope (Frankfurt, Germany). Digitized confocal images were processed with ImageJ image processing and analysis software.

#### RNA isolation, cDNA synthesis and RT-PCR analysis

Total RNA was extracted from iPSCs using Trizol Reagent (Thermo Fisher Scientific) according to the manufacturer’s protocol. For qPCR, 2 μg of total RNA was reverse transcribed with the iScript cDNA Synthesis Kit (Bio-Rad), according to the manufacturer’s protocol. qPCR was performed using the iTAQ Universal SYBER Green Supermix (Bio-Rad) according to the manufacturer’s instructions. The cycling profile for real-time PCR (40 cycles) was as follows: 30 s at 95 °C for enzyme activation, 5 s at 95 °C for initial denaturation, 5 s at 65 °C for annealing/extension and a 5 s melt curve step at 65°C–95 °C. Gene analysis was performed with the Bio-Rad CFX Manager software (Bio-Rad). Relative gene expression is normalized relative to unstimulated cells and fold variation is GAPDH normalized. The primer sequences used are shown in Table 1.

**TABLE 1:** qRT-PCR Primers.

Sequence Notes	Bases	Sequence
AFP, HOMO_SAPIENS	25 20	TCT GCA TGA ATT ATA CAT TGA CCA C AGG AGA TGT GCT GGA TTG TC
HNF4A, HOMO_SAPIENS	21 21	GAT GTA GTC CTC CAA GCT CAC GCC ATC ATC TTC TTT GAC CCA
ALB, HOMO_SAPIENS	22 21	CAA CAG AGG TTT TTC ACA GCA T GAG ATC TGC TTG AAT GTG CTG
CYP3A4, HOMO_SAPIENS	23 26	ATC ATG TCA GGA TCT GTG ATA GC GGG AAA TAT TTT GTC CTA CCA TAA GG
SERPINA1, HOMO_SAPIENS	21 24	GAT GTG CTT CCT CTC CCA TAG CCA TTA CCC TAT ATC CCT TCT CCT
GATA4, HOMO_SAPIENS	18 19	TTG CTG GAG TTG CTG GAA GGA AGC CCA AGA ACC TGA A
GAPDH, HOMO_SAPIENS	22 19	TGT AGT TGA GGT CAA TGA AGG G ACA TCG CTC AGA CAC CAT G
KRT7, HOMO_SAPIENS	21 18	ACC ACA AAC TCA TTC TCA GCA GGT CAG CTT GAG GCA CTG
KRT19, HOMO_SAPIENS	20 20	TTG GTT CGG AAG TCA TCT GC AGC CAC TAC TAC ACG ACC AT
SLC10A1, HOMO_SAPIENS	22 20	ACT GGC TTT CAG AAT TGC TTT G GCT GCC ACA ACT GAA GAA AC
RXRA, HOMO_SAPIENS	18 21	GGA GGT GAG GGA GGA GTT GCA TGA GTT AGT CGC AGA CAT
PPARA, HOMO_SAPIENS	24 21	TTC TGT TCT TTT TCT GGA TCT TGC CAG GCT ATC ATT ACG GAG TCC
NR1I2, HOMO_SAPIENS	17 22	TTC CGG GTG ATC TCG CA ACT GGC TAT CAC TTC AAT GTC A
ACTB, HOMO_SAPIENS	17 17	CCT TGC ACA TGC CGG AG ACA GAG CCT CGC CTT TG
PXR, HOMO_SAPIENS	20 17	CGT TCT TCA CCG ACT TCC TC CTG GGC AAG CTC TGG AG
CYP1A2, HOMO_SAPIENS	19 19	CAG CTC TGG GTC ATG GTT G CCT CCT TCT TGC CCT TCA C
MYC, HOMO_SAPIENS	23 20	TCT TCC TCA TCT TCT TGT TCC TC TCC TCG GAT TCT CTG CTC TC
REXO1, HOMO_SAPIENS	22 20	TTG GAG GTA CAG AAC TTG AGA C CCC ACA GTC CAT CCT TAC AG
KLF4, HOMO_SAPIENS	20 20	GTT TAC GGT AGT GCC TGG TC AAG AGT TCC CAT CTC AAG GC
CYP3A5, HOMO_SAPIENS	19 17	CAC AGG GAG TTG ACC TTC A CCC ACA CCT CTG CCT TT
CYP3A7, HOMO_SAPIENS	23 20	CTA TAC AGA CCA TGA GAG AGC AC CAG AAC ACC AGA GAC CTC AA
CYP1A2, HOMO_SAPIENS	19 19	CAG CTC TGG GTC ATG GTT G CCT CCT TCT TGC CCT TCA C
SOX9, HOMO_SAPIENS	18 18	CTT CAG GTC AGC CTT GCC CAT GAG CGA GGT GCA CTC
RXRA, HOMO_SAPIENS	18 21	GGA GGT GAG GGA GGA GTT GCA TGA GTT AGT CGC AGA CAT
RXRA, HOMO_SAPIENS	20 19	AGC TCT GAG AAG TGT GGG AT GAC CTA CGT GGA GGC AAA C
PPARA, HOMO_SAPIENS	18 22	AAG CTG GTG AAA GCG TGT AGG ATA GTT CTG GAA GCT TTG G
NR1I2, HOMO_SAPIENS	21 24	AGC ATA GCC ATG ATC TTC AGG CCA TTA CTC TGA AGT CCT ACA TTG
CYP3A4, HOMO_SAPIENS	24 26	ATT CCA AGC TTC TTA AAA AGT CCA AGA TAA GTA AGG AAA GTA GTG ATG C
CYP1A2, HOMO_SAPIENS	19 20	AGA AGG GAA CAG ACT GGG A TCC ACA CCA GCC ATT ACA AC
CYP3A5, HOMO_SAPIENS	20 22	TGG ACT CTT CGC TGA TTT GG AAG GAA GAC TCA CAG AAC ACA G
NR1I2, HOMO_SAPIENS	19 19	TCT TTG GGT CTC ACC TCC A CTT TGC ACC GGA TTG TTC A

## Proteomics

### Protein extraction

Proteins were extracted using RIPA buffer (Fisher Scientific). After measuring the total protein concentration, 50ug of total proteins were aliquoted and precipitated by adding 8X volume of cold acetone and 1X volume of 100% trichloroacetic acid (TCA). Precipitated proteins were washed with cold acetone and air dried before reconstitution into 40 µL of 8 M urea in 50 mM Tris-HCl (pH 8). Reduction and alkylation of the cysteine residues were then performed with 10 mM DTT and 55 mM iodoacetamide, respectively, followed by adding 360 µL of 50 mM ammonium bicarbonate buffer to reduce the urea concentration to below 1 M. The protein samples were digested by adding Trypsin (Thermo Scientific) at a 1:20 ratio (w/w) and incubated at 37°C overnight. Digested protein samples were cleaned up using C-18 micro-spin plate (Harvard Apparatus) before LC-MS analysis.

#### LC-MS/MS analysis

The LC-MS analysis was performed using an Orbitrap Fusion tribrid mass spectrometer (Thermo Scientific) connected to an Ultimate 3,000 nano-UPLC system (Thermo Scientific). Briefly, the peptide samples were trapped and washed on Pepmap100 C^18^ trap (5μm, 0.3 × 5 mm) at 20ul/min using 2% acetonitrile in water (with 0.1% formic acid) for 10 min and then separated on a Pepman100 RSLC C18 column (2.0 μm, 75-μm × 150-mm) using a gradient of 2%–40% acetonitrile with 0.1% formic acid over 120 min at a flow rate of 300 nL/min and a column temperature of 40°C. Eluted peptides were introduced into Orbitrap Fusion MS via nano-electrospray ionization (nano-ESI) source with a temperature of 300°C and spray voltage of 2000 V. The peptides were analyzed by data-dependent acquisition (DDA) in positive mode using Orbitrap MS analyzer for precursor scan at 120,000 FWHM from 400 to 2000 m/z and ion-trap MS analyzer for MS/MS scans in top speed mode (3-s cycle time) with dynamic exclusion settings (repeat count one and exclusion duration 15 s). Higher-energy collisional dissociation (HCD) was used as a fragmentation method with a normalized collision energy of 32%. The raw MS and MS/MS spectra were processed using the Proteome Discoverer software by Thermo Scientific (v1.4) and searched against the Uniprot human database using the SequestHT search algorithm. The Percolator PSM validator algorithm was used to validate the peptide spectrum matching and estimate the false discovery rate to be <1% (q-Value) (Käll et al., 2007).

#### Proteomic data analysis

For normalization, statistical analysis, and pathway analysis of genetic and sex-Specific Protein, the peptide spectrum match (PSM) count for each identified protein in the LC-MS/MS search results was used as a semi-quantitative measure for protein expression level. The PSM count for each protein in a specific sample was first normalized using the sum of the PSM counts for all proteins in that sample. Then, the mean PSM count for the three replicates in each group was calculated for each protein and further used for statistical analysis. Protein content was compared between the different biological groups sEVs. EdgeR R package was used to perform trimmed mean normalization (TMM), then the difference for protein expression between the groups was analyzed. Proteins upregulated or downregulated with a p-value cutoff of 0.05 were considered differentially expressed for further analyses. Gene Ontology pathway analyses were conducted using the Database for Annotation, Visualization, and Integrated Discovery (DAVID) and FunRich on differentially expressed protein genes. Uniprot Knowledgebase (UniProtKB) protein descriptions and gene products were imported into DAVID and FunRich for statistical analyses and GO term annotation based on integrated biological, molecular, and cellular pathways of the differentially expressed proteins.

#### Bioinformatics analysis

##### Data visualization

Volcano plots and heat maps were generated using https://www.bioinformatics.com.cn/

#### Pathway analysis

A list of significantly differentially expressed proteins (p-value ≤0.05) was analysed using ShinyGo: a graphical gene-set enrichment tool for animals and plants (http://bioinformatics.sdstate.edu/go). The top signaling pathways of the different matured hepatocytes and iPSCs were compared to the control group.

#### String analysis

The STRING (Search Tool for the Retrieval of Interacting Genes/Proteins) database was used for the illustration of predicted interactions of identified proteins and neighbor genes. The proteins significantly differentially expressed in the different groups were processed in STRING version 12.0 (https://string-db.org/) to obtain medium-confidence interaction data (score ≥0.7). The PPI network was visualized using the Cytoscape 3.2.1 software (https://cytoscape.org/).

### Imaging

Phase contrast imaging was performed on the EVOS Cell Imaging System (Thermo Fisher Scientific).

### Statistical analysis

All differentiation experiments were carried out in triplicate (n = 3). Data are presented as mean ± SEM. Statistical significance was determined using two tailed Student’s t-test with p < 0.05 determined to be significant.

## Results

### Characterization of human induced pluripotent stem cells

**FIGURE 1:**
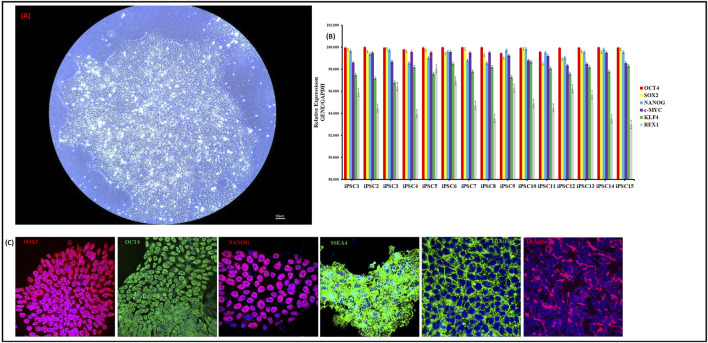
Characterization of human iPSCs. Phase Contrast Micrograph of one representative human iPSC line (20 ×). Images shown are for iPSC1 **(A)**. qRT-PCR analysis for relative expression of OCT-4, SOX-2, NANOG, cMYC, KLF4, and REX-4 **(B)**. Columns show the combined mean ΔΔC_t_ values for each marker. Data represent relative expression of transcripts normalized relative to GAPDH and expressed at the Mean ± SEM. Representative data from three independent experiments are shown. Immunostaining of human iPSC clones for pluripotency markers SOX2, OCT4, NANOG, SSEA-4, Tra-1–80 and Tra-1–60. Nuclei were stained with DAPI **(C)**. All 15 iPSC lines were stained and imaged.

Fifteen individual human iPSC lines were characterized and differentiated into HLCs with both the GF and SM protocol. A phase contrast micrograph showing typical iPSC colonies and morphology for the first iPSC1 line is shown in Figure 1A. Phase contrast micrographs for the other fourteen iPSC lines are shown in Supplementary Figure S1. Quantitative RT-PCR assessment of pluripotency and embryonic stem cell genes (OCT, SOX2, NANOG, c-MYC, KLF4 and REX1) is shown in Figure 1B. Embryonic stem cell and pluripotency proteins (SOX2, OCT4, NANOG, SSEA4, Tra-180, Tra-160) were assessed with immunocytochemistry (Figure 1C).

### Characterization of differentiated hepatocyte-like cells

**FIGURE 2:**
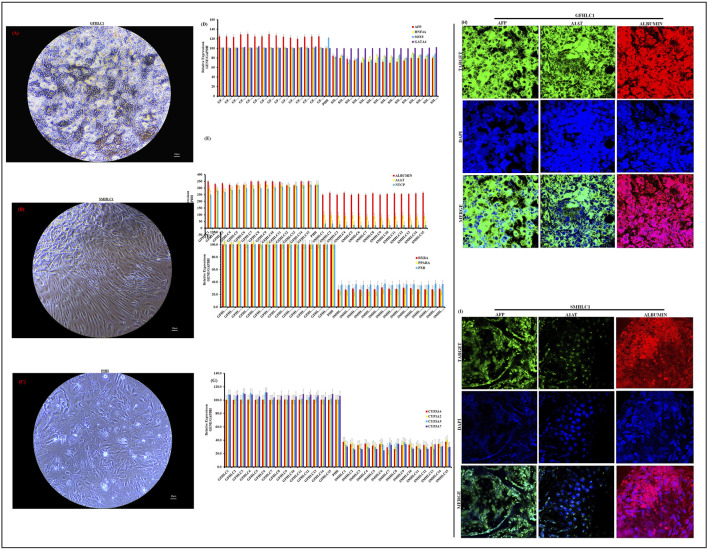
Characterization of Hepatocyte-like cell differentiation. Phase Contrast Micrographs of one representative differentiated iPSC cell line (iPSC1) (20 ×). Images shown are for GFHLC1 **(A)**, SMHLC1 **(B)** and PHH for comparison **(C)** (20 ×). qRT-PCR analysis for relative expression of hepatic genes in GFHLCs and SMHLCs **(D–G)**. Columns show the combined mean ΔΔC_t_ values for each marker. Data represents relative expression of transcripts normalized relative to GAPDH and undifferentiated controls. Data are represented as Mean ± SEM for three biologically independent experiments (n = 3). Immunostaining of hepatic protein expression in GFHLC1 **(H)** and SMHLC1 **(I)**. Representative data from three independent experiments are shown.

Human iPSCs were first differentiated into definitive endoderm (DE) cells, followed by hepatoblast specification and subsequent maturation into HLCs. We utilized our previously published SM approach (Asumda et al., 2018). The GF protocol was adapted from Xia Y et al. (Xia et al., 2017). We did not observe significant differences in the expression of DE genes between the SM and GF protocols (data not shown). SM hepatocyte maturation was completed with 100 nM of the SM mimetic N-hexanoic-Tyr, Ile-(6) aminohexanoic amide (dihexa). Dihexa is a potent HGF receptor agonist that promotes terminal differentiation of hepatoblasts (Siller et al., 2015; Mathapati et al., 2016; McCoy et al., 2013). At the end of 14 days of treatment with maturation media, we observed a stark difference in morphology of HLCs from the SM and GF protocols (Figure 2A, B; Supplementary Figure S1).

The GF protocol produced HLCs with a mature differentiated morphology. The GF-HLCs appear raised, polygonal shaped (6–12 sides) with well-defined refractile borders; the cytoplasm has a granular appearance with lipid droplets and/or vacuoles with multiple spherical nuclei or a large centrally located nucleus (Figure 2A; Supplementary Figure S1). The GF-HLCs cluster into compact aggregates with cord-like appearance with lucent rims suggestive of hepatic trabeculae and bile canaliculi. The surface topography of the GF-HLCs is suggestive of numerous microvilli and polarity (Figure 2A; Supplementary Figure S1). Commercially sourced PHHs utilized for these studies are depicted in (Figure 2C) for comparison. Our GF-HLCs are thus far not plateable; they cannot be passaged and re-plated on geltrex coated culture dishes after maturation. Attempts to passage our GF-HLCs result in cell death; they can however be maintained in culture after 14 days of maturation for an additional 2 weeks in Williams E Medium. Beyond this time frame, we observe significant cell death and disintegration. This suggests that GF-HLCs attain a level of hepatic differentiation that is synonymous with PHHs.

The SM protocol produced HLCs with the morphological appearance of established liver tumor derived cell lines (Figure 2B; Supplementary Figure S1). The SM-HLCs have a flattened appearance with fibroblast-like “ball and stick-like” protrusions. This morphology is suggestive of an immature and dedifferentiated phenotype. These cells have an abnormal rounding cell structure, with some cultures displaying bright lipid nodules but they lack the clearly demarcated polygonal shape of GF-HLCs or PHHs. They lack the cell surface characteristics of PHHs but have bright lipid droplets and/or vacuoles (Figure 2B; Supplementary Figure S1). SM-HLCs persist in culture for up to twenty passages and attach onto geltrex coated plates with each passage without appreciable cell death. This suggests high levels of dedifferentiation.

We compared the relative expression of key hepatocyte genes and transcription factors in GF-HLCs, SM-HLCs and PHHs. Expression of AFP, HNF4A, SOX9 and GATA4 is relatively similar in both GF-HLCs and SM-HLCs (Figure 2D). The expression profile of these genes in GF-HLCs is similar to PHHs. Relative expression of mature hepatocyte proteins ALBUMIN, α-1-antitrypsin and sodium taurocholate co-transporting polypeptide (ALBUMIN, A1AT, NTCP) were significantly higher in GF-HLCs and PHHs (Figure 2E). Modulators of hepatic energy, metabolic, lipid homeostasis, immune and inflammatory response (PPARA, RXRA, PXR) have a significantly higher expression in GF-HLCs and PHHs in comparison to SM-HLCs (Figure 2F). We assessed the relative expression of multiple components of the cytochrome P450 system (CYP450) including CYP3A4, CYP1A2, CYP3A5, and CYP3A7. Components of the CYP450 system are uniformly expressed at a significantly higher level in GF-HLCs and PHHs in comparison to SM-HLCs (Figure 2G). Immunostaining for AFP, ALBUMIN and A1AT proteins showed a significantly higher expression level in GF-HLCs in comparison to SM-HLCs (Figures 2H, I).

### Proteomics analysis of differentiated hepatocyte-like cells

**FIGURE 3:**
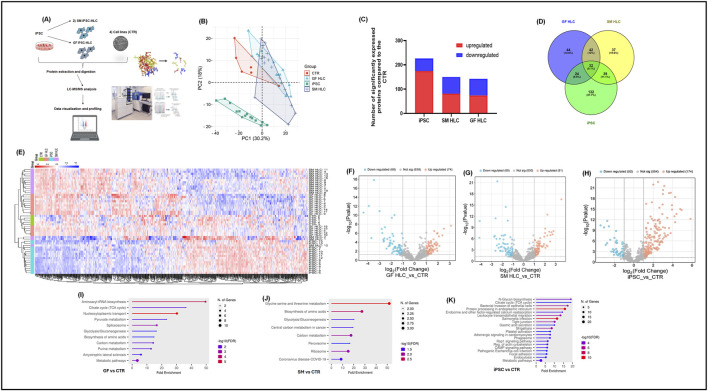
Proteomic analysis of iPSCs, GF HLCs, and SM HLCs compared to Controls (CTR). Diagrammatic presentation of the mass spectrometry workflow used in this study **(A)**. Principal component analysis (PCA) showing the various cell types: CTR, iPSc, GFHLCs, SMHLCs as different shapes. The CTR is represented in red, iPSC in green, GFHLCs in light blue, and SMHLCs in dark blue **(B)**. The PCA plot was generated using peptide abundance data of all peptides analysed per cell type with 10 replicates. A bar graph showing the number of significantly upregulated (red) and downregulated (blue) proteins in each cell type compared to the CTR **(C)**. A Venn diagram illustrating the unique differently expressed proteins in each condition was generated **(D)**. Comparative heatmap of all the replicates per cell type (iPSC, CTR, GFHLC and SMHLC) and the identified protein groups **(E)**. Proteomic pathways analysis of significantly differentially expressed proteins. Volcano plots of the differentially expressed proteins from the different cell types: iPSC, GFHLCs, SMHLCs **(F–H)**. The negative x-axis represents downregulation (blue) in cell type and the positive axis represents upregulated (red) proteins in the different cell types **(F–H)**. Dot plots were generated using the uniquely differentially expressed proteins in ShinyGO analysis, with KEGG pathway enrichment and fold enrichment based on the number of genes present in each pathway **(I–K)**. The FDR cut-off was set at 0.05, and the number of pathways was set to 20.

The proteomes of iPSCs, GF-HLCs, SM-HLCs and controls (CTR) (HUH7, HEP3B, HEPG2, SNU398, PHHs) were analyzed by data-dependent acquisition mass spectrometry (DDA-MS) (Figure 3A). A principal component analysis (PCA) was able to differentiate the proteome of the four groups. The PCA showed significant overlap between GF-HLC, SM-HLC and CTR groups, compared to the iPSCs dataset (Figure 3B). The heatmap showed a global change in upregulated and downregulated proteins from the undifferentiated state (iPSC) to the differentiated state (GF-HLCs, SM-HLCs, CTR) (Figure 3E). The significant differentially expressed proteins (DEPs) between the four data groups, were identified. A minimum fold change ≥1.5 and maximum false discovery rate (FDR) adjusted p-value (q-value) ≤0.05 was used to filter proteins that were significantly different between the iPSC, GF-HLCs and SM-HLCs compared to CTR. The iPSC group has the greatest number of DEPs (226), in comparison to SM-HLCs (150) and GF-HLCs (142) (Figure 3C). Only, 9.1% of significant DEPs identified were common between iPSCs, GF-HLCs, and SM-HLCs, when compared to the CTR (Figure 3D). The DEPs were separated into up and downregulated DEPs and visualized using volcano plots (Figures 3F–H). To understand the variation in DEPs between groups, we categorized the DEPs with ShinyGO analysis based on the specific biological pathways they affect. The GF-HLCs and SM-HLCs had similarly enriched pathways (Figures 3I, J). These pathways include carbon metabolism, glycolysis/gluconeogenesis and the biosynthesis of amino acids pathways (Figures 3I, J). The iPSCs, when compared to the CTR were enriched in protein processing and cytoskeletal re-organization pathways (Figure 3K).

**FIGURE 4:**
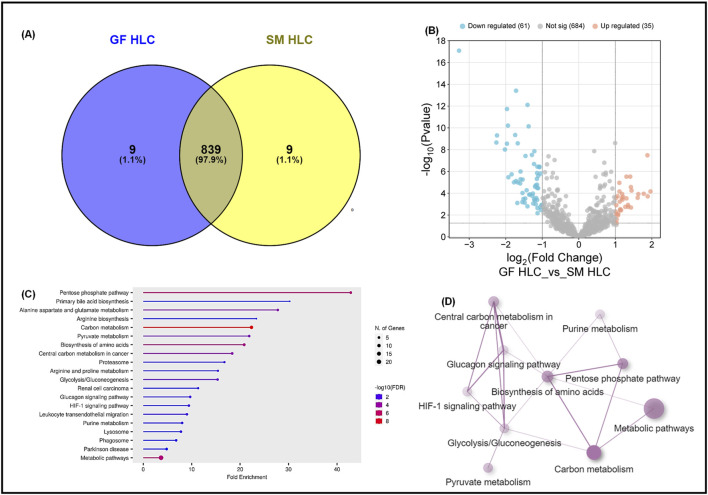
Proteomic analysis of GF hepatocytes compared to SM hepatocytes. A Venn diagram illustrating the total number of proteins in GFHLCs *versus* SMHLCs **(A)**. Volcano plots of the differentially expressed proteins in GFHLCs and SMHLCs **(B)**. The negative x-axis represents downregulation (blue) in GFHLCs compared to SMHLCs, and the positive axis represents upregulated (red) proteins in the different GFHLCs compared to SMHLCs. Dot plot generated using the differentially expressed proteins in ShinyGO analysis, with KEGG pathway enrichment and fold enrichment based on the number of genes present in each pathway **(C)**. The FDR cut-off was set at 0.05, and the number of pathways was set to 20. String analysis Network analysis of the differentially expressed proteins corresponding to the different pathways using STRING network (Fold cut-off set to 0.7) **(D)**.

We conducted a direct comparison of the whole proteome of the GF-HLC and SM-HLC datasets. HLCs derived from the GF and SM protocols have a 97.8% similarity in the total number of proteins between the two data sets (Figure 4A). Despite having a high similarity of the total number of proteins identified in each data set, the expression levels of these proteins were significantly different (p value ≤0.05). Of the common proteins (839), there were 61 significantly downregulated proteins and 35 significantly upregulated proteins in the GF-HLCs compared to SM-HLCs (Figure 4B). Proteomic analysis showed that these significantly differentially expressed proteins are involved primarily in metabolic pathways including carbon metabolism, amino acid biosynthesis, pyruvate metabolism, glucagon signaling, pentose phosphate pathway, HIF-1 signaling and glycolysis/gluconeogenesis (Figure 4C). Further analysis using ShinyGO and STRING networks showed that these metabolic pathways are connected (Figure 4D). This suggests that a primary difference between the GF and SM protocol in terms of their individual effect on iPSCs during differentiation is the extent of metabolic rewiring and maturation in the HLCs.

## Discussion

In this study, we compared a growth factor (GF) and small molecule (SM) protocol for differentiation of human iPSCs into hepatocyte-like cells (HLCs). We previously published on the SM protocol (Asumda et al., 2018). Our experience with the SM approach has been that it produces HLCs with a dedifferentiated phenotype. DMSO is a well-established SM that has been utilized for enhancing and maintaining hepatocyte differentiation (De La Vega and Mendoza-Figureueroa, 1991; Dubois-Pot-Schneider et al., 2022; Su and Waxman, 2004). Recent improvements to the SM strategy have included ammonium chloride (NH4Cl), FH1 (*N*,*N*'-(Methylenedi-4,1-phenylene)bis-acetamide), FPH1 (2-(N-(5-chloro-2-methylphenyl) methylsulfonamido)-N-(2,6-difluorophenyl) acetamide), the Activin/NODAL/TGF-β pathway inhibitor (A8301; 3-(6-methylpyridin-2-yl)-N-phenyl-4-quinolin-4-ylpyrazole-1-carbothioamide) (Gao et al., 2020; Du et al., 2018; Pan et al., 2022). These additions to the SM cocktail are geared towards hepatocyte function enhancement and maturation. Dihexa is a key component of the SM approach; it is a potent HGF receptor agonist that promotes terminal differentiation of hepatoblasts and hepatic maturation (Siller et al., 2015; Mathapati et al., 2016; Gao et al., 2020; Du et al., 2018; Pan et al., 2022; McCoy et al., 2013). The most consistently published growth factors include Activin A, Wnt3a, Hepatocyte Growth Factor (HGF), Oncostatin M (OSM), fibroblast growth factor 4 (FGF4), and bone morphogenic protein 4 (BMP4) (Xia et al., 2017; Carpentier et al., 2016; Si-Tayeb et al., 2010; Lv et al., 2024; Chen et al., 2012; Sullivan et al., 2010; Hannan et al., 2013; Jafarpour et al., 2018). Low dose dexamethasone, which is the key maturation component of the GF protocol, is known to promote a mature hepatic morphology and architecture; it enhances expression of liver-enriched transcription factors and proteins (Olsavsky Goyak et al., 2010; Arterburn et al., 1995; Nelson et al., 2013). Our data demonstrates that the GF protocol consistently produces HLCs with a more mature hepatic phenotype. The significant variability of SM-HLC morphology across multiple cell lines (Supplementary Figure S1) is likely an issue of reproducibility of the SM protocol across multiple cell lines. It may also be a by-product of differences in individual cell-line response to the small molecules. We aim to undertake further mechanistic studies that will illuminate the morphology variation among the small molecule-derived HLCS of the different iPS cells.

Alpha-fetoprotein (AFP) is a glycoprotein that is highly expressed in embryonic liver and in hepatic progenitor cells (hepatoblasts) (Olsavsky Goyak et al., 2010). The elevated level of AFP in mature liver is associated with pathological processes including tumorigenesis and with hepatocyte dedifferentiation (Olsavsky Goyak et al., 2010). Although the adult liver is composed primarily of hepatocytes (∼80%), it is heterogenous with other types of cells present (endothelial, biliary and stellate cells) (Usynin and Panin, 2008; Aizarani et al., 2019; Olsavsky Goyak et al., 2010). In this study, both the GF and SM protocols produced a hepatic cell population that is heterogenous with a sizeable persistent population of hepatoblasts or cells with fetal characteristics in the final maturation stage. This is demonstrated by the persistently high expression of AFP observed with qRT-PCR, and immunocytochemistry analysis across all fifteen iPSC lines. Culture conditions determine whether *in vitro* cultured hepatocytes obtain and maintain a mature differentiated state *versus* a proliferative dedifferentiated phenotype. Depending on the goals and specific needs for iPSC-HLCs as an *in vitro* model system, a dedifferentiated state might be preferable, for example, during investigation of liver regeneration mechanisms. However, for studies of metabolism, biotransformation, and viral infection, *in vitro* HLCs must have a level of differentiation that is as close to PHHs or *in vivo* liver as possible; in which case, GF-HLCs are preferrable. Our goal is to refine the GF protocol to produce a more homogeneous HLC population at the end of maturation.

Using a DDA MS-based quantitative proteomic approach, a comparative analysis of the whole proteome of GF-HLCs and SM-HLCs was compared to a control group consisting of liver tumor-derived carcinoma cell lines and PHHs. The different cell types (iPSCs, GF-HLCs, SM-HLCs and CTR) were analyzed by principal component analysis (PCA) which showed a significant overlap in the global protein abundance in the GF-HLC, SM-HLC and CTR. However, the GF-HLC, SM-HLC and CTR together have a unique hepatic proteome profile that is distinct from iPSCs. This observation is consistent with the fact that GF-HLCs, SM-HLCs and CTR cells are all liver derived. We observed a much higher level of variation in the SM-HLCs over the fifteen different cell lines in comparison to the GF-HLCs, which is suggestive of a more consistent and reproducible GF protocol. It also suggests consistent response to the growth factors irrespective of the iPSC line. The consistency of the GF protocol is further supported by the uniform morphology of all fifteen GF-HLCs. Both protocols produce a heterogenous population of hepatic cells at the end of maturation but based on immunocytochemical analysis, the GF protocol produces a significantly higher percentage of HLCs.

**FIGURE 5:**
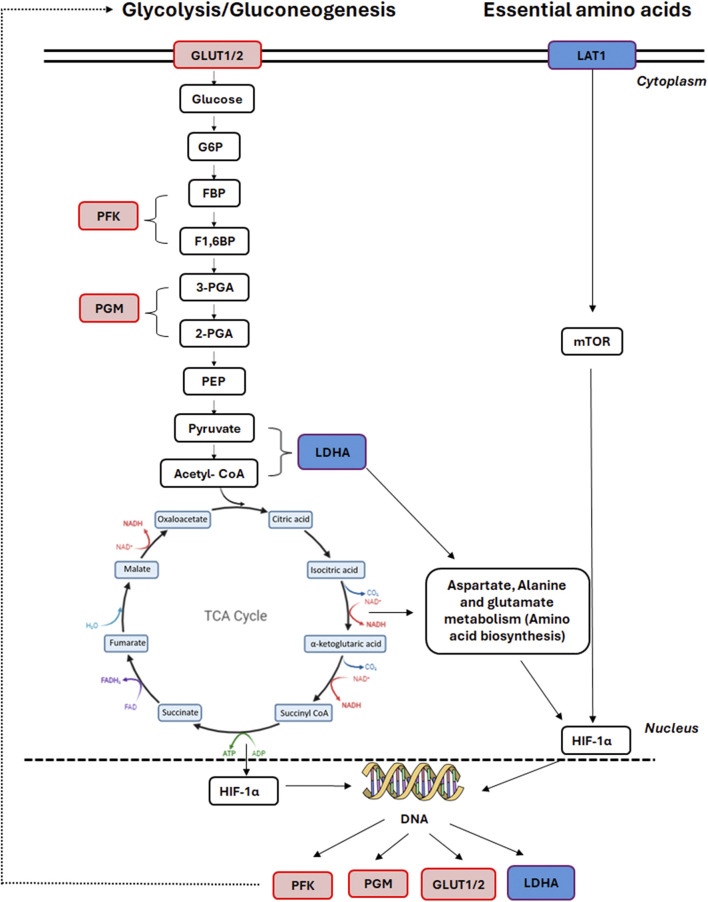
Affected TCA cycle proteins in GF HLCs compared to SM HLCs*.* Graphical representation of the differentially expressed proteins in the central carbon metabolism pathway. Upregulated proteins are represented in red and downregulated proteins are represented in blue.

Both protocols had a similar number of significantly differentially expressed proteins (DEPs) compared to the control group and shared three commonly enriched pathways: carbon metabolism, glycolysis/gluconeogenesis and the biosynthesis of amino acids pathways. Proteomic analysis of the GF-HLCs and SM-HLCs showed that the DEPs are involved in carbon metabolism, amino acid biosynthesis, pyruvate metabolism, glucagon signaling, pentose phosphate pathway, HIF-1 signaling and glycolysis/gluconeogenesis. Comparison of metabolic and energy pathways in HLCs from the two protocols to the established liver tumor-derived cell lines, confirmed our *in vitro* culture observation that SM-HLCs are more like established liver tumor-derived cell lines. Studies of established liver tumor cell lines (HepG2 and Huh7) show that these cells have significantly reduced levels of mature liver proteins in comparison to healthy hepatocytes (Wiśniewski et al., 2016). Liver tumor-derived cell lines are inexpensive, easier to culture and have stable enzyme concentrations when compared to PHHs. However, they have low or absent expression levels of drug-metabolizing enzymes, impaired host defense system, and loss of the NTCP receptor (Clayton et al., 2005; Brandon et al., 2003; Choi et al., 2009). Our analysis shows that GF-HLCs have significantly increased expression of glycolytic intermediates: glucose transporter-1and 2 (GLUT1/2), 6-phosphofructo-1 kinase (PFK), and phosphoglycerate mutase (PGM) (Figure 5). This suggests that GF-HLCs utilize glucose as the primary metabolic fuel; PHHs utilize either glucose and/or fatty acids. The SM-HLCs have significantly enriched glycerin, serine and threonine metabolism and have increased TCA cycle intermediates and lactate dehydrogenase-1 (LDH1) expression (Figure 5). The serine/glycerin pathway is an energy requiring biosynthetic pathway that requires glucose (Kalhan and Hanson, 2012). Serine and glycine provide precursors for proteins, nucleic acids, lipids and TCA intermediates (Possemato et al., 2011; Renwick et al., 1998; Tedeschi et al., 2013; Pan et al., 2021). Upregulation of serine/glycine metabolism correlates with increased proliferative capacity in cancer cells and dedifferentiation (Todisco et al., 2019; Ballester et al., 2019; Wiśniewski et al., 2016; Yang et al., 2024; Sajnani et al., 2017; Fekir et al., 2019; Zhang et al., 2019; Ciccarone et al., 2017; Amelio et al., 2014). Our observations in this study, and the resulting data suggest that the SM-HLCs have a similar *in vitro* phenotype to the liver tumor-derived cell lines. The likely explanation for this finding is that like tumor-derived cells, the SM-HLCs have deregulated consumption of glucose and amino acids; they select glycerin/serine over glucose as the primary metabolic fuel. It is also likely the SM-HLCs have alterations in metabolic gene expression with resultant changes of TCA cycle/glycolytic/mitochondrial intermediates that are geared towards high proliferation, high plasticity and dedifferentiation. This is likely the effect of the specific culture components.

## Conclusion

In summary, we demonstrate that both the GF and SM protocols generate iPSC-HLCs with definitive hepatic phenotype. The data shows that the GF derived HLCs have a more mature adult phenotype that is most similar to PHHs. The lack of passaging capacity in the GF-HLCs is a limitation for certain applications but this mature phenotype makes the GF-HLCs ideal for *in vitro* studies of metabolism, biotransformation, and viral infection. The SM derived HLCs have *in vitro* characteristics of liver tumor-derived cell lines. These SM HLCs maintain a de-differentiated phenotype and for this reason, they are more appropriate for potentially replacing damaged hepatocytes and for supporting liver regeneration *in vivo* or for *in vitro* studies geared at understanding liver regeneration. Their de-differentiated phenotype suggests a potential to survive, engraft and trans-differentiate once transplanted into the liver. It also makes them a more appropriate model for bio-artificial liver and whole liver bioengineering (Forbes and Newsome, 2012; Telles-Silva et al., 2024).

## Data Availability

The original contributions presented in the study are included in the article/supplementary material, further inquiries can be directed to the corresponding author.

## Abbreviations

hiPSC, Human induced pluripotent stem cells; HLC, hepatocyte-like cells; PHH, primary human hepatocytes; GF, growth factors; SM, small molecules; A1AT, α1-antitrypsin; ALB, ALBMIN.
