# Supplementary figures and images for: Comparative analysis of small molecule and growth factor-derived human induced pluripotent stem cell-derived hepatocyte-like cells
Source: Front Cell Dev Biol. 2025 Jun 26;13:1594340. doi: 10.3389/fcell.2025.1594340 (PMC12240953; doi:10.3389/fcell.2025.1594340)

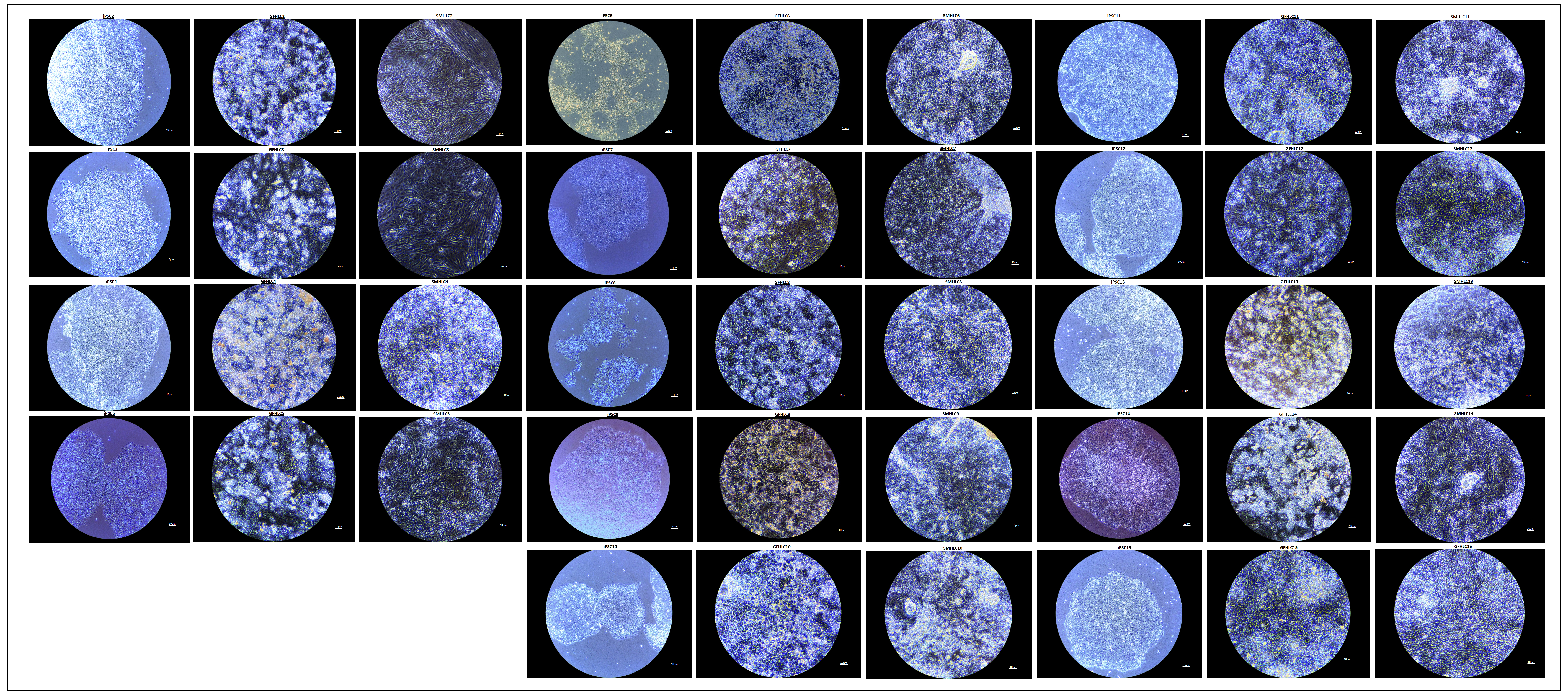

Supplement: Supplementary file 1 [file Image1.jpeg]
